# A qualitative analysis of an A*β*-monomer model with inflammation processes for Alzheimer’s disease

**DOI:** 10.1098/rsos.231536

**Published:** 2024-05-15

**Authors:** Ionel Ciuperca, Laurent Pujo-Menjouet, Leon Matar-Tine, Nicolas Torres, Vitaly Volpert

**Affiliations:** ^1^ CNRS, Ecole Centrale de Lyon, Université Jean Monnet, Universite Claude Bernard Lyon 1, ICJ UMR5208, INSA Lyon, Lyon, Villeurbanne 69622, France; ^2^ Departamento de Matemática Aplicada, Universidad de Granada, Granada, Andalusia, Spain; ^3^ Peoples’ Friendship University of Russia, Moscow 117198, Russia

**Keywords:** Alzheimer’s disease, persistence, bifurcation analysis, hysteresis, inflammation

## Abstract

We introduce and study a new model for the progression of Alzheimer’s disease (AD) incorporating the interactions of A *β *-monomers, oligomers, microglial cells and interleukins with neurons through different mechanisms such as protein polymerization, inflammation processes and neural stress reactions. To understand the complete interactions between these elements, we study a spatially homogeneous simplified model that allows us to determine the effect of key parameters such as degradation rates in the asymptotic behaviour of the system and the stability of equilibrium. We observe that inflammation appears to be a crucial factor in the initiation and progression of AD through a phenomenon of hysteresis with respect to the oligomer degradation rate d . This means that depending on the advanced state of the disease (given by the value of the A β -monomer degradation rate d : large value for an early stage and low value for an advanced stage) there exists a critical threshold of initial concentration of interleukins that determines if the disease persists or not in the long term. These results give perspectives on possible anti-inflammatory treatments that could be applied to mitigate the progression of AD. We also present numerical simulations that allow us to observe the effect of initial inflammation and monomer concentration in our model.

## 1. Introduction

Understanding the origin and development of Alzheimer’s disease (AD) has been a challenging problem for biologists during the past few decades. As in many neurodegenerative diseases, AD is known to be associated with the misconformation, aggregation and propagation of different proteins in the nervous system [1–5]. They form stable oligomers that eventually accumulate in the so-called amyloid plaques and this phenomenon is believed to lead to a progressive irreversible neuronal damage. One of these proteins that appears to be relevant in the early stages of the development of AD are the A β -monomers, whose precise mechanisms of aggregation and diffusion are yet to be discovered.

In this context, mathematical models arise as a useful approach to understand the different processes underlying AD. Several types of models have been considered, including from simple systems of ordinary differential equations to more complex partial differential equations, such as transport equations [6], reaction–diffusion models [7–10] and stochastic control models [11].

The goal of this article is to understand the complete interactions between A β -monomers, oligomers, microglial cells and interleukins through a new system of partial differential equations, involving the development of AD in the brain. Neurons produce A β -monomers that almost instantaneously start to polymerize into proto-oligomers. In this aggregation process, proto-oligomers are able to polymerize or depolymerize and once they reach a critical size they become stable under the form of A β -oligomers. These latter are assumed to be totally stable in the sense that neither polymerization nor depolymerization is possible for A β -oligomer equilibrium [12,13]. This mechanism on A β -oligomers is known as the amyloid cascade hypothesis and there is a general consensus that it is a key factor in the progression of AD [1,5].

Besides the mechanism of polymerization, oligomers interact with microglial cells, considered as auxiliary cells in the nervous systems regulating brain development. They induce an inflammation reaction through a chemical cascade in microglial cells, releasing interleukins [14,15]. These interleukins then activate an increase of A β -monomer production from the neurons. However, if the concentration of A β -oligomers is high enough, then a reaction of stress called unfolded protein response (UPR) [4] is triggered which leads to a decrease of A β -monomer production, while the rest of oligomers diffuse in the neuronal environment. In this context, two opposed mechanisms of stimulation and inhibition will determine the persistence of AD or not.

Moreover, oligomers are brought and displaced by microglia to the amyloid plaques, that is, an aggregate of A β -oligomers that becomes an inert element (no diffusion, no polymerization, no depolymerization). Each element of the system (monomers, proto-oligomers, and oligomers except those in the amyloid plaques) diffuses, with a size-dependent rate. Microglial cells can also have random motility, but they displace free A β -oligomers to the amyloid plaques through a chemotactic process and amyloid plaques will more likely develop where the concentration of microglial cells is high. These cells are known indeed to be very reactive to neuronal insults [16–19].

Inflammation processes seem to be crucial to control the disease progression [15] and to find possible therapeutic strategies to mitigate the negative effects of AD. For example, it is suggested by Rivers-Auty *et al*. [20] that diclofenac-based drugs might be associated with slower cognitive decline with possible perspectives on AD progression. However, despite epidemiological evidence, robust clinical trials have not been successful in providing efficacy evidence of such anti-inflammatory treatments [21–23]. On the other hand, in Ali *et al*. and Imbimbo *et al*. [24,25], it is suggested that anti-inflammatory treatments might be effective if they are applied years before the development of clinical symptoms. Furthermore, in Imbimbo *et al*. [25], it is mentioned that some anti-inflammatory treatments decrease the levels of A β by allosterically inhibiting the γ -secretase complex, which could give interesting perspectives in finding efficient cures. Other treatment suggestions include actions on multiple targets besides neuroinflammatory and neuroprotective effects such as anti-amyloid and anti-tau effects [26,27].

Bertsch *et al*. and Andrade-Restrepo *et al*. [9,10], using reaction–diffusion type equations, describe the initiation and progression of AD under the hypothesis of amyloid cascade where the A β in its oligomeric form is toxic for neurons. In our paper, in addition to the amyloid cascade hypothesis, we take into account the effect of inflammation on the progression of the disease. This inflammation appears through the process of recruitment of microglial cells and then the activation of interleukins (IL-1). As a general goal, we aim to understand the progression of AD through an analysis-compatible simplified version of this base model.

**Figure 1.**
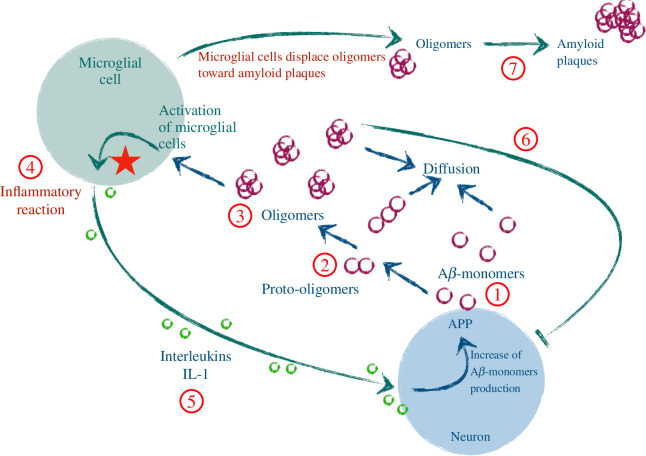
Schematic representation of A β -monomers and inflammation cycle. Neurons produce A β -monomers (1) that polymerize into proto-oligomers (2). These proto-oligomers eventually reach a critical size to become stable oligomers (3). They activate microglial cells triggering an inflammatory reaction (4) by producing interleukins. The interleukins stimulate neurons (5) to increase A β -monomer production, closing the positive feedback cycle. Moreover, when oligomer concentration is high, neurons are stressed (6) and decrease the A β -monomer production, while oligomers are displaced by microglial cells towards the amyloid plaques (7).

The article is organized as follows. In §2, we introduce the main system of partial differential equations and we describe the reactions involving monomers, (proto-)oligomers, microglial cells and interleukins, which are summarized in figure 1. Then, in §3, we deal with a spatially homogeneous version of the main model, where polymerization and depolymerization processes are simplified. For this simplified model, we analyse the existence of steady states depending on the parameters. Finally, in §4, we present numerical simulations of the simplified model in order to observe the different possible dynamics of solutions and the stability of the steady states.

## 2. Mathematical model

Let us detail each equation of the system. In this model, we consider that dynamics occur in a part of the brain considered as an open bounded domain $\Omega \subset R^{d}$ (with d∈{2,3} ) and the main variables of the system are given in table 1.

**Table 1.** Variables of the mathematical model.

variable	definition
$u_{i}(t,x)$	concentration of A β -proto-oligomers of size i
u(t,x)	concentration of A β -oligomers
$u_{p}(t,x)$	concentration of oligomers in the amyloid plaques
m(t,x)	concentration of A β -monomers
M(t,x)	concentration of microglial cells
I(t,x)	concentration of interleukins

### 2.1. Proto-oligomers

A β -proto-oligomers have a size ranging from i=2 to $i=i_{0}-1$ and become oligomers when they reach the size $i=i_{0}$ (see point (2) in figure 1). Equations for proto-oligomers with size $i=[2,\cdots ,i_{0}-1]$ are given by


$$\frac{\partial u_{i}}{\partial t}(t,x)=r_{i-1}u_{i-1}(t,x)m(t,x)+b_{i}u_{i+1}(t,x)-r_{i}u_{i}(t,x)m(t,x)-bu_{i}(t,x)+\nu_{i}\Delta u_{i}(t,x),$$

where $r_{1}$ is the bi-monomeric nucleation rate (with the notation $u_{1}=m(t,x)$ the monomer concentration), $b_{i}$ is the rate of monomer loss from proto-oligomers and $r_{i}$ is the rate of monomer gain. The couple $\left(r_{i},b_{i}\right)$ , $i\in [2,\cdots ,i_{0}-1]$ is called kinetic coefficients with the notation $b_{i}=b$ if $i\leq i_{0}-2$ and $b_{i_{0}-1}=0$ .

The first term of the right-hand side stands on the one hand for the bi-monomeric nucleation when i=2 and on the other hand for the polymerization with rate $r_{i-1}$ ( i≥3 ) of a proto-oligomer of size i−1 with the contact of a monomer, giving then a proto-oligomer of size i . The second term describes the depolymerization with rate $b_{i}$ of a proto-oligomer of size i+1 to a proto-oligomer of size i . The third and fourth terms are related to the symmetric process respectively of polymerization and depolymerization of a proto-oligomer of size i . Finally, each proto-oligomer can diffuse with a size-dependent coefficient (the smaller the size, the faster the diffusion).

### 2.2. Free oligomers

The variation of the A β -oligomer population is described as follows (see point (3) in figure 1):


$$\begin{array}{lll} \frac{\partial u}{\partial t}(t,x) & = & r_{i_{0}-1}u_{i_{0}-1}(t,x)m(t,x)-\gamma (M(t,x))u(t,x)-\tau_{0}u(t,x)+\nu_{i_{0}}\Delta u(t,x), \end{array}$$

where the first term of the right-hand side stands for the polymerization with rate $r_{i_{0}-1}$ of a proto-oligomer of size $i_{0}-1$ with the contact of a monomer giving then an oligomer of size $i_{0}$ . The second term describes the recruitment of oligomers to the amyloid plaques by microglial cells M with a rate γ given by


$$\gamma (M)=\gamma_{0}+\frac{\gamma_{1}M}{1+\gamma_{2}M},$$

depending on M through a Michaelis–Menten function with parameters $\gamma_{i}$ ( i∈{0,1,2} ) and the third term corresponds to the degradation of oligomers with rate $\tau_{0}$ . Finally, each oligomer diffuses with rate $\nu_{i_{0}}$ . It is important to remember here that oligomers neither polymerize nor depolymerize, unlike proto-oligomers.

### 2.3. Oligomers in the amyloid plaques

The variation of the A β -oligomer population stuck in the amyloid plaques is described as follows (see point (7) in figure 1):


$$\begin{array}{l} \frac{\partial u_{p}}{\partial t}(t,x)=\gamma (M(t,x))u(t,x)-\tau_{p}u_{p}(x,t), \end{array}$$

where the first term of the right-hand side stands for the recruitment of free oligomers to the amyloid plaques by microglial cells M with a rate γ and the second term represents the corresponding loss with rate $\tau_{p}$ . We remind here that oligomers in the amyloid plaques neither polymerize, depolymerize nor diffuse.

### 2.4. Monomers

The variation of the A β -monomer population is described as follows (see point (1) in figure 1):


$$\begin{array}{lll} \frac{\partial m}{\partial t}(t,x) & = & -r_{1}m^{2}-\sum_{i=2}^{i_{0}-1}r_{i}u_{i}(t,x)m(t,x)+b\sum_{i=2}^{i_{0}-1}u_{i}(t,x)\\ & & +S(u(t,x),I(t,x))-d\;m(t,x)+\nu_{1}\Delta m(t,x), \end{array}$$

where I(t,x) is the concentration of interleukins and the function S is given by


(2.1) $$S(u,I)=\frac{\tau_{S}}{1+Cu^{n}}I,\;n\geq 1.$$

The term S(u,I) is called the stress function. According to the form of this function, under a high concentration of oligomers u surrounding the neuron, this latter will be stressed and stop the production of A β -monomers, which means that S(u,I) is close to 0 (see point (6) in figure 1).

We remark that the neuron can be torn between the decision of producing A β -monomers due to the inflammation (caused by the interleukins) and the stress caused by the amount of oligomers surrounding the neurons causing the UPR process that stops this A β production. Note that this object is one of the major key properties in our model. For simplicity, we do not take into account the fact that microglia produce A β -monomers and this will be considered in a future work with a more complex model.

The first and second terms of the right-hand side stand, respectively, for the bi-monomeric nucleation and the polymerization of proto-oligomers of all sizes, while the third term describes the corresponding processes of depolymerization of proto-oligomers. The fourth term is the source term depending on the inflammation reaction caused by the interaction of A β -oligomers with microglial cells. The fifth term describes the degradation of the monomers with a rate d . This rate d may depend on oligomer concentration and behave as a Hill function, but for simplicity, we consider in the sequel that d is a given positive constant. Finally, the last term stands for the monomer diffusion ability with rate $\nu_{1}$ .

### 2.5. Microglial cells

The evolution of the microglial cell population is described as follows (see point (4) in figure 1):


$$\begin{array}{lll} \frac{\partial M}{\partial t}(t,x) & = & D_{1}\Delta M(t,x)-\alpha \nabla \cdot (M(t,x)\nabla u(t,x))\\ & & +\lambda_{M}+\frac{\alpha_{1}u(t,x)}{1+\alpha_{2}u(t,x)}\left(\hat{M}-M(t,x)\right)M(t,x)-\sigma M(t,x), \end{array}$$

where the first term of the right-hand side stands for the diffusion of microglial cells with rate $D_{1}$ . The second term represents the chemotaxis of microglial cells in response to the increase of oligomer population. This chemotactic effect results in an activation of microglial cells due to the presence of oligomers which causes an inflammatory reaction with the production of interleukins (IL-1). The third term describes the proliferation of microglial cells at a constant rate $\lambda_{M}$ . In the fourth term, $\hat{M}$ is the maximum capacity of microglial cells in the neuron environment and the last term characterizes the loss of microglial cells with rate σ .

### 2.6. Interleukins

The equation for the evolution of interleukins is (see point (5) in figure 1)


$$\frac{\partial I}{\partial t}(t,x)=D_{I}\Delta I(t,x)+\frac{\tau_{1}u(t,x)}{1+\tau_{2}u(t,x)}M(t,x)-\tau_{3}I(t,x),$$

where the first term of the right-hand side is the diffusion of the interleukins, and the second term represents the proliferation which depends on the concentration of oligomers through a Michaelis–Menten function with parameters $\tau_{1},\tau_{2}$ and the microglial cells. The third term represents the loss of interleukins with rate $\tau_{3}$ .

We note that all equations are complemented with Neumann boundary conditions with zero flux through ∂Ω and the parameters of the system are non-negative real numbers. The main interactions of this system are summarized in figure 1.

## 3. A bi-monomeric simplified model

In order to proceed to a full mathematical analysis, and understand the qualitative dynamics of the actors of this problem, we consider a simplified model version of the full system of partial differential equations. We assume a bi-monomeric nucleation, that is, two monomers can merge to form a free oligomer ( m+m→u ) and the intermediate proto-oligomer phase is absent. For this case, we assume that when a monomer attaches to a free oligomer, the latter does not change and the monomer is consumed ( u+m→u ). The equations of the simplified PDE system are the following:


(3.1) $$\{\begin{array}{ll} \frac{\partial u}{\partial t}=\nu_{2}\Delta u+r_{1}m^{2}-\gamma (M)u-\tau_{0}u, & \\ \frac{\partial u_{p}}{\partial t}=\gamma (M)u-\tau_{p}u_{p}, & \\ \frac{\partial m}{\partial t}=\nu_{1}\Delta m+\frac{\tau_{S}}{1+Cu^{n}}I-dm-r_{2}um-r_{1}m^{2},\\ \frac{\partial M}{\partial t}=D_{1}\Delta M-\alpha \nabla \cdot (M\nabla u)+\frac{\alpha_{1}u}{1+\alpha_{2}u}(\hat{M}-M)M-\sigma M+\lambda_{M},\\ \frac{\partial I}{\partial t}=D_{I}\Delta I+\frac{\tau_{1}u}{1+\tau_{2}u}M-\tau_{3}I. \end{array}$$

We also assume that when a monomer binds to an oligomer, then the monomer is consumed with rate $r_{2}$ and the number of oligomer molecules does not change. Under these assumptions, we notice that there is no term involving the rate $r_{2}$ in the equation of oligomers.

We could recall here that it is essential to consider all intermediate stages of oligomer formation, as they are important and could potentially play a significant role in the disease dynamics. However, we chose to study a simplified problem initially to conduct a thorough analysis of stability and highlight the phenomenon of hysteresis (see §4.1). This decision could, however, be biologically explained by the fact that the polymerization and depolymerization process is much faster compared with the degradation process, as was considered in previous works [10,11] with the choice of parameter values. Therefore, we consider the intermediate stages at equilibrium. This is obviously a significant simplification, and in our future work, we will incorporate all stages into a more comprehensive study.

### 3.1. Spatially homogeneous model

In addition to the previous subsection, and to simplify the analysis in this work, we focus on spatially homogeneous solutions of the bi-monomeric model equation (3.1). We recall that this present work has two main objectives: (i) to introduce the most comprehensive possible model, which, in our view, is the most biologically realistic; and (ii) to propose an initial simplification to provide clear insights into a novel dynamics of this process. Of course, this simplification comes at the expense of tissue realism, especially spatial heterogeneity. The biological interpretation of the choice of homogeneity here is to localize the disease in a specific tissue (a region of the brain), where anti-inflammatory signals would have a notable effect. Naturally, in our future works, we will explore much more heterogeneous regions with potentially richer dynamics, but much more challenging to obtain and interpret.

For simplicity, we assume that the rate of recruitment of oligomers to the amyloid plaques γ(M) is constant, which corresponds essentially to considering an average rate of oligomers being recruited and we consider that oligomers have a highly stable structure and their degradation is negligible, which means $\tau_{0}=0$ . However, the results of the qualitative analysis of the system do not change if we consider the degradation of oligomers. Under this setting, the model is reduced to the following system of ordinary differential equations:


(3.2) $$\{\begin{array}{l} \frac{du}{dt}=r_{1}m^{2}-\gamma_{0}u,\\ \frac{du_{p}}{dt}=\gamma_{0}u-\tau_{p}u_{p},\\ \frac{dm}{dt}=\frac{\tau_{S}}{1+Cu^{n}}I-dm-r_{2}um-r_{1}m^{2},\\ \frac{dM}{dt}=\frac{\alpha_{1}u}{1+\alpha_{2}u}(\hat{M}-M)M-\sigma M+\lambda_{M},\\ \frac{dI}{dt}=\frac{\tau_{1}u}{1+\tau_{2}u}M-\tau_{3}I. \end{array}$$

Because of this simplification, we obtain the following result.

**Proposition 3.1.** *For any non-negative initial condition* $\left(u_{0},u_{p0},m_{0},M_{0},I_{0}\right)$ *, the system has a unique global solution which is bounded.*

*Proof*. Existence and uniqueness of a local solution are straightforward from the Cauchy–Lipschitz theorem for ordinary differential equations. For the positivity of solutions, consider the vector field $F=(f_{1},\ldots ,f_{5})$ for $y=(y_{1},\ldots ,y_{5})\in R^{5}$ given by


$$\{\begin{array}{l} f_{1}(y)=r_{1}y_{3}^{2}-\gamma_{0}y_{1},\\ f_{2}(y)=\gamma_{0}y_{1}-\tau_{p}y_{2},\\ f_{3}(y)=\frac{\tau_{S}}{1+Cy_{1}^{n}}I-dy_{3}-r_{2}y_{1}y_{3}-r_{1}y_{3}^{2},\\ f_{4}(y)=\frac{\alpha_{1}y_{1}}{1+\alpha_{2}y_{2}}(\hat{M}-y_{4})y_{4}-\sigma y_{4}+\lambda_{M},\\ f_{5}(y)=\frac{\tau_{1}y_{1}}{1+\tau_{2}y_{2}}y_{3}-\tau_{3}y_{5}, \end{array}$$

and observe that F satisfies the quasi-positivity property, that is, for all indices i∈{1,…,5} we have


$$\forall (y_{j}{)}_{j\neq i}\in (R^{+}{)}^{4},\;f_{i}(y_{1},\ldots ,y_{i-1},0,y_{i+1},\ldots ,y_{5})\geq 0.$$

Thus, from proposition 2.1 in Haraux [28], we conclude that the solution remains non-negative because of this property.

We now assert that the solution remains bounded. Indeed, from the fourth equation of system (3.2), we conclude that if M is large enough then dM/dt<0 and, therefore, M(t) remains bounded. By reapplying the same argument, we subsequently conclude the same result for the rest of the variables of the system. Since the solutions of system (3.2) are bounded, they are defined for all t>0 . ∎

### 3.2. Steady states

The stationary points of system (3.2) correspond to solutions of the following system:


(3.3) $$\{\begin{array}{l} \;\;\;\;\;\;\;r_{1}m^{2}-\gamma_{0}u=0,\\ \;\;\;\;\;\;\;\;\gamma_{0}u-\tau_{p}u_{p}=0,\\ \frac{\tau_{S}}{1+Cu^{n}}I-dm-r_{2}um-r_{1}m^{2}=0,\\ \frac{\alpha_{1}u}{1+\alpha_{2}u}(\hat{M}-M)M-\sigma M+\lambda_{M}=0,\\ \;\;\;\;\;\;\frac{\tau_{1}u}{1+\tau_{2}u}M-\tau_{3}I=0. \end{array}$$

One of the solutions of this system is the disease-free equilibrium, given by $\left(0,0,0,\frac{\lambda_{M}}{\sigma },0\right)$ . Besides this equilibrium, there may be other steady states depending on the parameter values of our system, whose existence will be studied in this section. Concerning the disease-free equilibrium, we get the following result.

**Proposition 3.2.** *For the system* *(3.2)* *, the disease-free equilibrium* $\left(0,0,0,\frac{\lambda_{M}}{\sigma },0\right)$ *is locally asymptotically stable for every choice of positive parameters.*

*Proof*. The Jacobian matrix around the vector $\left(0,0,0,\frac{\lambda_{M}}{\sigma },0\right)$ is given by


$$J=\left[\begin{array}{ccccc} -\gamma_{0} & 0 & 0 & 0 & 0\\ \gamma_{0} & -\tau_{p} & 0 & 0 & 0\\ 0 & 0 & -d & 0 & \tau_{S}\\ \alpha_{1}\left(\hat{M}-\frac{\lambda_{M}}{\sigma }\right)\frac{\lambda_{M}}{\sigma } & 0 & 0 & -\sigma & 0\\ \tau_{1}\frac{\lambda_{M}}{\sigma } & 0 & 0 & 0 & -\tau_{3} \end{array}\right],$$

whose set of eigenvalues is given by $\left\{-\gamma_{0},-\tau_{p},-d,-\sigma ,-\tau_{3}\right\}$ . Since they are all negative, then the disease-free equilibrium is locally asymptotically stable.∎

An interesting question is to determine under which parameter values the existence of non-trivial steady states (i.e. AD persists) holds. In this regard, we have the following result.

**Theorem 3.3.** *Assume that the parameters satisfy the condition*


(3.4) $$\sigma \gamma_{0}\tau_{3}<\tau_{1}\tau_{S}\lambda_{M}.$$

*Then for* d>0 *small enough, there exist at least two positive steady states of system* *(3.3)* *. If* d>0 *is large enough, then there are no positive solutions of system* *(3.3)* *, regardless of condition* *(3.4)* *.*

*Proof*. From system (3.3), we solve for u and $u_{p}$ in terms of m and we get the following relation:


$$u=\rho m^{2},\;u_{p}=\frac{r_{1}}{\tau_{p}}m^{2}\;\text{with}\;\rho =\frac{r_{1}}{\gamma_{0}}.$$

From the equation of microglial cells, we solve the quadratic equation of M in terms of u and by taking the positive root we get the following equality:


(3.5) $$M=\frac{\sqrt{\Delta (u)}-\sigma -(\sigma \alpha_{2}-\hat{M}\alpha_{1})u}{2\alpha_{1}u},$$

with $\Delta (u)=(\sigma +(\sigma \alpha_{2}-\hat{M}\alpha_{1})u{)}^{2}+4\lambda_{M}\alpha_{1}u(1+\alpha_{2}u)$ . For the interleukins we get the relation


$$I=\frac{\tau_{1}}{\tau_{3}}\frac{\rho m^{2}}{1+\tau_{2}\rho m^{2}}M.$$

Substituting these expressions into the equation of m in equation (3.3), we obtain the equation with respect to m :


(3.6) m(P(m)+d)=mF(m),

where the functions *P* and *F* are given by


(3.7) $$\begin{array}{rl} P(m) & =r_{2}\rho m^{2}+r_{1}m,\\ F(m) & =\frac{2\tau_{1}\tau_{S}\lambda_{M}}{\tau_{3}}\frac{\rho m(1+\alpha_{2}\rho m^{2})}{\left[\sqrt{\Delta (\rho m^{2})}+\sigma +(\sigma \alpha_{2}-\hat{M}\alpha_{1})\rho m^{2}](1+\tau_{2}\rho m^{2})(1+C\rho^{n}m^{2n}\right)}. \end{array}$$

The disease-free equilibrium corresponds to the case when m=0 in equation (3.6) . In order to get a positive steady state of system (3.2), we must determine the values where P(m)+d=F(m) .

From the definition of Δ(u) , we remark that the denominator is strictly positive in the function F . We observe that F(0)=0 , F(m)>0 for m>0 and F(m)→0 as m→∞ , since the numerator is of order $O(m^{3})$ and the denominator is of order $O(m^{2n+4})$ .

Moreover $F^{\prime }(0)$ is given by


$$F^{\prime }(0)=\frac{r_{1}\tau_{1}\tau_{S}\lambda_{M}}{\sigma \gamma_{0}\tau_{3}}>0.$$

From condition (3.4), we observe that


$$P^{\prime }(0)<F^{\prime }(0),$$

hence there exists $\tilde{m}>0$ such that


(3.8) $$P(m)<F(m)\;\text{for all}\;m\in (0,\tilde{m}).$$

Let us denote $m_{0}=\sup\;\{\tilde{m}>0:\;\text{property holds}\}>0$ in equation (3.8). Since P(m)→∞ as m→∞ , we conclude that $m_{0}<\infty$ and from continuity, we get


(3.9) $$P(m)<F(m)\;\text{for all}\;m\in (0,m_{0}),\;P(m_{0})=F(m_{0}).$$

Let us now denote


$$\tilde{d}=\max_{y\in [0,m_{0}]}(F(y)-P(y)),$$

which is strictly positive by condition (3.9). Let $y_{0}\in (0,m_{0})$ such that $F(y_{0})-P(y_{0})=\tilde{d}$ . We now take an arbitrary *d* such that $0<d<\tilde{d}$ . And the following inequalities hold:


$$P(0)+d>F(0),\;P(y_{0})+d<F(y_{0}),\;P(m_{0})+d>F(m_{0}).$$

Therefore, there exists a positive solution of equation (3.6) in $\left(0,y_{0}\right)$ and another positive solution in $\left(y_{0},m_{0}\right)$ . This proves the existence result.

For the non-existence result, observe that F reaches a maximum, since F(0)=0 and F(m)→0 as m→∞ , and this maximum is independent of d . Hence, for d large enough, we have that


$$P(m)+d>\max_{y>0}F(y)\geq F(m)\;\text{for all}\;m\geq 0,$$

and we conclude that there is no solution in that case. ∎

From the previous result, we assert that when the rest of the parameters are fixed, there exists a critical value of degradation rate of monomers $d=d_{c}$ , such that for $d>d_{c}$ the system (3.2) has only the disease-free equilibrium and for $d<d_{c}$ there are at least two positive solutions. From a biological point of view, this means that a high degradation of monomers can avoid the persistence of AD, while a lower degradation of monomers is not sufficient to stop the pathogenic cycle of monomers, oligomers and interleukins.

## 4. Numerical simulations

The main goal of this section is to present a qualitative analysis of the possible asymptotic behaviours and the stability of steady states of system (3.2) through a bifurcation diagram with respect to the degradation rate of monomers and the concentration of interleukins at equilibrium. From the previous analysis of §3, the key parameters for the existence of positive steady states where the disease persists are the degradation rates. For these simulations, we rely on the parameter values given in table 2.

**Table 2.** Parameter values for the numerical simulations of equation (3.2).

parameter	value	units	description
$r_{1}$	$10^{-1}$	$l\;(\text{mol}{)}^{-1}\;(\text{months}{)}^{-1}$	bi-monomeric polymerization rate
$r_{2}$	$10^{-1}$	$l\;(\text{mol}{)}^{-1}\;(\text{months}{)}^{-1}$	polymerization rate of monomers attaching to oligomers
d	variable	$(\text{months}{)}^{-1}$	degradation rate of monomers
$\gamma_{0}$	$5\times 10^{-2}$	$(\text{months}{)}^{-1}$	recruitment rate of oligomers to the amyloid plaques
$\tau_{1}$	1	$l\;(\text{mol}{)}^{-1}\;(\text{months}{)}^{-1}$	growth coefficient of interleukins
$\tau_{2}$	1	$l\;(\text{mol}{)}^{-1}$	growth coefficient of interleukins
$\tau_{3}$	1	$(\text{months}{)}^{-1}$	degradation rate of interleukins
$\tau_{p}$	$3\times 10^{-2}$	$(\text{months}{)}^{-1}$	degradation rate of oligomers in the amyloid plaques
$\tau_{S}$	1	$(\text{months}{)}^{-1}$	coefficient of neural stress
C	1	$l^{n}\;(\text{mol}{)}^{-n}$	coefficient of stress function
n	2	—	power coefficient of stress function
$\alpha_{1}$	1	$l^{2}\;(\text{mol}{)}^{-2}\;(\text{months}{)}^{-1}$	growth coefficient of microglial cells
$\alpha_{2}$	1	$l\;(\text{mol}{)}^{-1}$	growth coefficient of microglial cells
$\lambda_{M}$	$10^{-3}$	$\text{mol}\;l^{-1}\;(\text{months}{)}^{-1}$	rate of proliferation of microglial cells
$\hat{M}$	1		capacity of microglial cells
σ	$10^{-3}$	$(\text{months}{)}^{-1}$	degradation rate of microglial cells

The values are chosen with the order of magnitude between 10^−3^ and 1, in the typical range of a biological process. The values can be re-scaled if needed, but the qualitative behaviour is similar. In particular, we assumed that the polymerisation process of monomers is faster than the corresponding degradation of monomers and oligomers.

To the best of our understanding, there is limited knowledge available regarding the values of the parameters associated with this model. Indeed, each experiment pertaining to a specific aspect of our study has been conducted under varying conditions, rendering the precise determination of data values seemingly meaningless. Notably, even defining a biological range proves challenging. Nevertheless, through in-depth discussions with collaborators in the field of biology, it becomes apparent that the paramount consideration lies in the ratio between each key parameter. For example, regardless of polymer size, polymerization rates should be approximately the same. Similarly, the degradation rate for oligomers in plaques is significantly smaller than any other degradation rate. Since we are interested in the qualitative behaviour of system (3.2), modifying the values in table 2 leads essentially to the same type of results, and the parameters can be re-scaled and re-normalized as was presented in the numerical simulations in Andrade-Restrepo *et al*. [10] for the polymerization process.

### 4.1. Effect of inflammation

The results of theorem 3.3 motivate the analysis of the steady states as a function of the degradation rate of monomers *d*. In particular, we are interested in the inflammation processes that lead to the persistence of AD. In this context, we analyse the bifurcation diagram for the concentration of interleukins at equilibrium $I^{*}$ depending on the degradation rate of monomers d as the bifurcation parameter. The rest of the components of a steady state of equation (3.2) are calculated according to the system (3.3).

**Figure 2.**
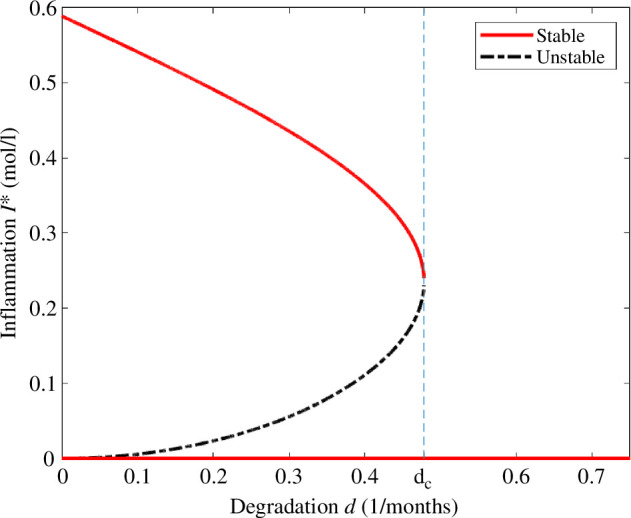
Bifurcation diagram of the steady states for the concentration of interleukins $I^{*}$ in terms of the degradation rate of monomers *d* with the parameters of table 2. The disease-free equilibrium exists for all values of d>0 and it is stable. For $d<d_{c}$ , we have two other non-trivial equilibria, where the maximal one is stable and the other one is unstable. For $d>d_{c}$ , we get no positive steady states.

We observe in figure 2 that for all d>0 the disease-free equilibrium is asymptotically stable. Moreover, there exists a critical degradation rate of monomers $d_{c}\approx 0.4779\;(\text{months}{)}^{-1}$ , which we call the critical degradation rate of persistence, such that for $d<d_{c}$ there exists two positive steady states where the maximal one is asymptotically stable and the other one is linearly unstable. If $d>d_{c}$ then the disease-free steady state is the only equilibrium of the system.

From the bifurcation diagram of figure 2, we observe the importance of the degradation rate of monomers, which determines the existence of steady states where AD persists. We also observe that for a small degradation rate d , the concentration of interleukins at equilibrium $I^{*}$ is large.

The bifurcation analysis is quite challenging even for the simplified version of the model. Thus, we proceed to numerical simulations in the next section in order to show the asymptotic behaviour of solutions of system (3.2) under different degradation rates of monomers *d* and initial data. In particular, we choose the initial values given in table 3.

**Table 3.** Initial data for the numerical simulations of equation (3.2).

parameter	value	units	description
$u_{0}$	$10^{-4}$	mol l^−1^	concentration of free oligomers
$u_{p0}$	0	mol l^−1^	concentration of oligomers in the amyloid plaques
$m_{0}$	$10^{-3}$	mol l^−1^	concentration of monomers
$M_{0}$	1	mol l^−1^	concentration of microglial cells
$I_{0}$	variable	mol l^−1^	concentration of interleukins

This means that we study system (3.2) under a small initial concentration of monomers and free oligomers. We also consider that oligomers in the amyloid plaques are initially absent, while microglial cells are already developed. We vary the initial concentration of interleukins $I_{0}$ and the degradation rate of monomers *d* to study the asymptotic behaviour of equation (3.2).

**Figure 3.**
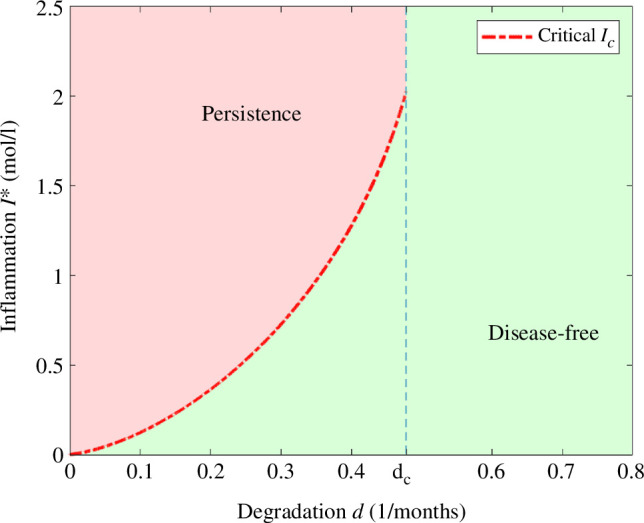
Asymptotic behaviour of solutions in terms of degradation rate of monomers *d* and the initial inflammation $I_{0}$ . For the parameter values from table 2 and initial data from table 3, we get the critical threshold of inflammation $I_{c}$ and the critical degradation rate $d_{c}$ . For $d<d_{c}$ , we get that AD persists for $I_{0}>I_{c}$ and does not persist if $I_{0}<I_{c}$ . If $d>d_{c}$ the disease does not persist.

In figure 3, we present the possible asymptotic behaviours of system (3.2) in terms of the degradation rate of monomers *d* and the initial inflammation $I_{0}$ with the parameters in table 2 and initial data in table 3.

In particular, we observe a phenomenon of hysteresis for $d<d_{c}$ , where $d_{c}$ is the critical degradation rate in figure 2, which implies the existence of a critical threshold value for the inflammation $I_{c}>0$ (depending on the rest of the parameters and the initial data) that determines if AD persists or not. We observe in figure 3 that for degradation rates of monomers satisfying $d<d_{c}$ , solutions of equation (3.2) converge to the disease-free equilibrium when $I_{0}<I_{c}$ and converge to the positive stable equilibrium when $I_{0}>I_{c}$ .

Moreover, for small values of *d,* a small initial concentration of interleukins $I_{0}$ suffices for the persistence of AD, while for values close to the critical degradation rate of persistence $d_{c}$ , a higher initial concentration of $I_{0}$ is needed. Furthermore, for $d<d_{c}$ most solutions converge either to the disease-free equilibrium or to the stable positive equilibrium. This global stability result is to be proven in future work. When $d>d_{c}$ , in the absence of positive steady states, we conjecture that all the solutions of system (3.2) converge to the disease-free equilibrium.

Next, we show some numerical simulations of solutions of the simplified system (3.2) in order to illustrate the effects of hysteresis and inflammation processes in the convergence to a steady state.

**Figure 4.**
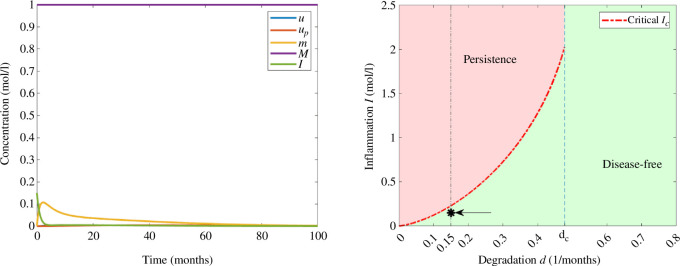
Example 1. (Left) Numerical solution of system (3.2) with $d=0.15\;(\text{months}{)}^{-1}$ and $I_{0}=0.15\;{mol\;l}^{-1}$ . The parameters correspond to those in table 2 and the initial data in table 3. (Right) Asymptotic behaviour in terms of degradation rate of monomers *d* and the initial inflammation $I_{0}$ . The value of $I_{0}$ is indicated with an arrow and *d* by a vertical line.

For a small degradation rate, $d=0.15\;(months{)}^{-1}$ , we observe from figure 2 that we have three steady states, and by choosing $I_{0}=0.15\;{mol\;l}^{-1}$ , we observe in figure 4 that the solution converges to the disease-free equilibrium. In this example, the concentration of interleukins is decreasing and the threshold of inflammation is not reached. Moreover, the concentrations of monomers increase until reaching the maximum value and eventually decrease and the concentrations of free oligomers and oligomers in the amyloid plaques remain relatively low.

**Figure 5.**
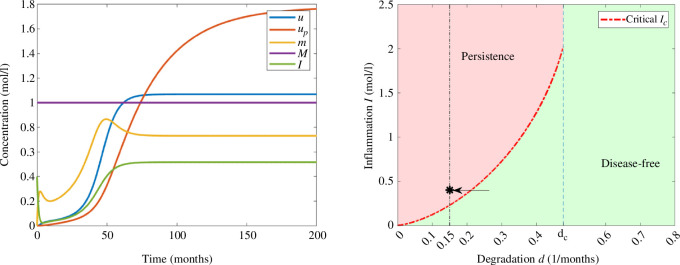
Example 2. (Left) Numerical solution of system (3.2) with $d=0.15\;(\text{months}{)}^{-1}$ and $I_{0}=0.4\;{mol\;l}^{-1}$ . The parameters correspond to those in table 2 and the initial data in table 3. (Right) Asymptotic behaviour in terms of degradation rate of monomers *d* and the initial inflammation $I_{0}$ . The value of $I_{0}$ is indicated with an arrow and *d* by a vertical line.

If we increase the value of initial inflammation to $I_{0}=0.4\;{mol\;l}^{-1}$ in figure 5, the solution converges to the stable positive steady state, since the critical threshold value $I_{c}$ is less than $I_{0}$ . In this example, the concentrations of free oligomers and oligomers in the amyloid plaques are increasing towards the corresponding values of equilibrium. Inflammation is initially decreasing until it reaches the minimum value and eventually increases towards the equilibrium value, while the concentration of monomers has increasing and decreasing phases due to the effect of stress mechanisms, nucleation and degradation.

**Figure 6.**
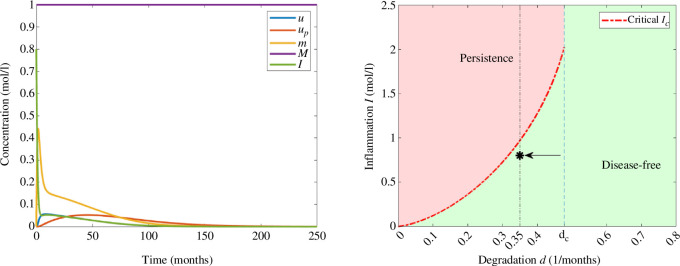
Example 3. (Left) Numerical solution of system (3.2) with $d=0.35\;(\text{months}{)}^{-1}$ and $I_{0}=0.8\;{mol\;l}^{-1}$ . The parameters correspond to those in table 2 and the initial data in table 3. (Right) Asymptotic behaviour in terms of degradation rate of monomers *d* and the initial inflammation $I_{0}$ . The value of $I_{0}$ is indicated with an arrow and *d* by a vertical line.

In a similar way for a larger degradation rate $d=0.35\;(\text{months}{)}^{-1}$ , we have also three steady states according to figure 2. For $I_{0}=0.8\;{mol\;l}^{-1}$ , we observe in figure 6 that the solution converges to the disease-free equilibrium. In this example, the concentration of interleukins is eventually decreasing, since the threshold of inflammation is not reached. Moreover, the concentrations of monomers, free oligomers and oligomers in the amyloid plaques increase until they reach their corresponding maximum values and eventually decrease. In particular, the maxima are higher compared with those observed in figure 4.

**Figure 7.**
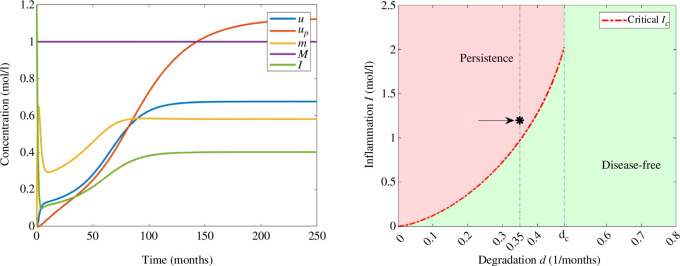
Example 4. (Left) Numerical solution of system (3.2) with $d=0.35\;(\text{months}{)}^{-1}$ and $I_{0}=1.2\;{mol\;l}^{-1}$ . The parameters correspond to those in table 2 and the initial data in table 3. (Right) Asymptotic behaviour in terms of degradation rate of monomers *d* and the initial inflammation $I_{0}$ . The value of $I_{0}$ is indicated with an arrow and *d* by a vertical line.

For $I_{0}=1.2\;{mol\;l}^{-1}$ , the solution converges to the positive stable steady state in figure 7, leading to the persistence of AD since the critical threshold value $I_{c}$ is less than $I_{0}$ . Similar to figure 5, the concentrations of free oligomers and oligomers in the amyloid plaques are increasing towards the corresponding values of equilibrium. Inflammation is initially decreasing until it reaches the minimum value and eventually increases towards the equilibrium value, while the concentration of monomers has increasing and decreasing phases due to the effect of stress mechanisms, nucleation and degradation. Moreover, we observe that equilibrium values are lower than those observed in figure 5 since the degradation rate of monomers is higher.

**Figure 8.**
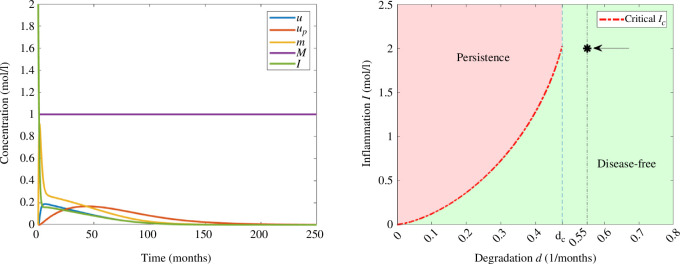
Example 5. (Left) Numerical simulations of system (3.2) for the parameters in table 2 and the initial data in table 3 with $d=0.55\;(\text{months}{)}^{-1}$ and $I_{0}=2\;{mol\;l}^{-1}$ . (Right) Asymptotic behaviour in terms of degradation rate of monomers d and the initial inflammation $I_{0}$ . The value of $I_{0}$ is indicated with an arrow and d by a vertical line.

Finally, for $d=0.55\;(\text{months}{)}^{-1}$ , we get only the trivial steady state according to figure 2, so that for $I_{0}=2\;{mol\;l}^{-1}$ , the solution converges to the disease-free equilibrium as we see in figure 8. The behaviour of concentrations is similar to that in figure 6.

In this bi-stable case, the solutions of system (3.2) converge to the positive stationary equilibrium if the initial concentrations of interleukins, monomers or free oligomers are sufficiently large. The phenomenon of hysteresis could indicate that AD can be initiated by the inflammation.

### 4.2. Effect of monomer concentration

Similarly to the analysis of inflammation in the persistence of AD, we study the effect of the initial concentration of monomers. In this context, we present some numerical simulations to illustrate the same hysteresis phenomenon with respect to the initial concentration of monomers. We choose the initial values given in table 4.

**Table 4.** Initial data for the numerical simulations of equation (3.2).

parameter	value	units	description
$u_{0}$	0	mol l^−1^	concentration of free oligomers
$u_{p0}$	0	mol l^−1^	concentration of oligomers in the plaques
$m_{0}$	variable	mol l^−1^	concentration of monomers
$M_{0}$	1	mol l^−1^	concentration of microglial cells
$I_{0}$	0	mol l^−1^	concentration of interleukins

This means that we study system (3.2) under a given concentration of oligomers while free oligomer, oligomers in the plaques and interleukins are initially absent. As in the previous analysis of §4.1, we assume that microglial cells are already developed. We vary the initial concentration of interleukins $m_{0}$ and the rate of monomers d to show the asymptotic behaviour of equation (3.2). We remark that similar results are obtained if we take a positive initial concentration of free oligomers and monomers are initially absent.

**Figure 9.**
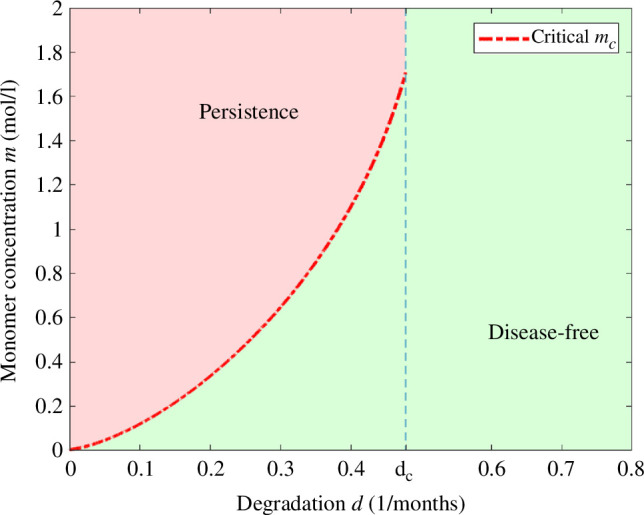
Asymptotic behaviour of solutions in terms of degradation rate of monomers d and the initial concentration of monomers $m_{0}$ . For the parameter values of table 2 and initial data of table 4, we get the critical threshold of monomer concentration $m_{c}$ and the critical degradation rate $d_{c}$ . For $d<d_{c}$ we get that AD persists for $m_{0}>m_{c}$ and does not persist if $m_{0}<m_{c}$ . If $d>d_{c}$ the disease does not persist.

In figure 9, we present the possible asymptotic behaviours of system (3.2) in terms of the degradation rate of monomers d and the initial concentration of monomers $m_{0}$ , with the parameters of table 2 and initial data of table 4, following the same analysis presented in figure 3.

Similarly to §4.1, we observe the same phenomenon of hysteresis for $d<d_{c}$ , where $d_{c}$ is the critical degradation rate in figure 2, which implies the existence of the respective critical threshold value for the initial concentration of monomers $m_{c}>0$ (depending on the rest of parameters and the initial data), that determine if AD persists or not. We observe in figure 9 that for degradation rates of monomers satisfying $d<d_{c}$ , solutions of equation (3.2) converge to the disease-free equilibrium when $m_{0}<m_{c}$ and converge to the positive stable equilibrium when $m_{0}>m_{c}$ .

**Figure 10.**
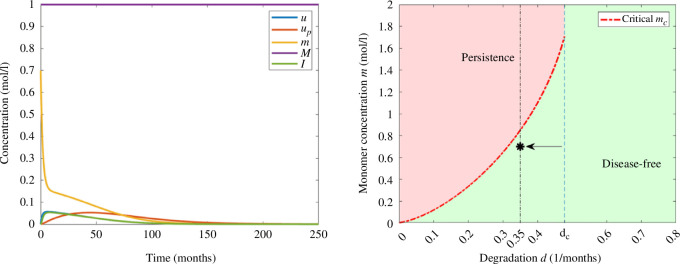
Example 6. (Left) Numerical solution of system (3.2) with $d=0.35\;(\text{months}{)}^{-1}$ and $m_{0}=0.7\;{mol\;l}^{-1}$ . The parameters correspond to those in table 2 and the initial data in table 4. (Right) Asymptotic behaviour in terms of degradation rate of monomers d and the initial concentration of monomers $m_{0}$ . The value of $m_{0}$ is indicated with an arrow and d by a vertical line.

For $d=0.35\;(\text{months}{)}^{-1}$ and $m_{0}=0.7\;{mol\;l}^{-1}$ , we observe in figure 10 that the solution converges to the disease-free equilibrium. In this example, the concentration of monomers is decreasing (contrary to the case of the interleukins in §4.1), due to its intrinsic degradation rate and the formation of free oligomers. Moreover, the concentrations of free oligomers, oligomers in the amyloid plaques and interleukins increase until they reach their corresponding maximum values and eventually decrease in the same way as in the previous examples.

**Figure 11.**
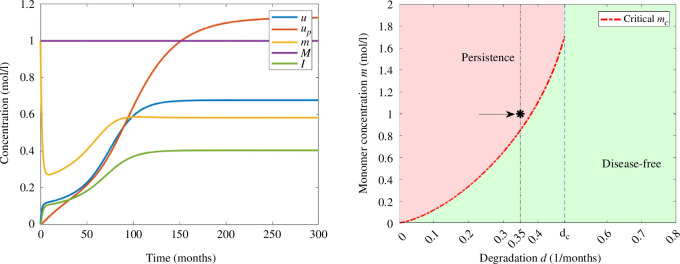
Example 7. (Left) Numerical solution of system (3.2) with $d=0.35\;(\text{months}{)}^{-1}$ and $m_{0}=1\;{mol\;l}^{-1}$ . The parameters correspond to those in table 2 and the initial data in table 4. (Right) Asymptotic behaviour in terms of degradation rate of monomers d and the initial concentration of monomers $m_{0}$ . The value of $m_{0}$ is indicated with an arrow and d by a vertical line.

For the same value of the degradation rate d and $I_{0}=1\;{mol\;l}^{-1}$ , we observe in figure 11 that the solution converges to the positive stable steady state, since the critical threshold value $m_{c}$ is less than $m_{0}$ . In this example, the concentrations of free oligomers, oligomers in the amyloid plaques and interleukins are increasing towards the corresponding values of equilibrium. The monomer concentration initially decreases until it reaches the minimum value and eventually increases towards the equilibrium value.

From figures 10 and 11, we observe essentially the same phenomenon of hysteresis and asymptotic behaviour as for the simulations in §4.1.

## 5. Discussion and perspectives

From the previous numerical simulations of the bi-monomeric model equation (3.2) in §4, and even if it corresponds to a simplified version of the original model, we already get a first qualitative approach in understanding the influence of inflammation and the degradation rates in the persistence of AD through a phenomenon of hysteresis, which determines the asymptotic behaviour of solutions of system (3.2) through a critical threshold for the inflammation in terms of the parameters and the initial data. This qualitative analysis suggests that AD may be triggered by an initial high concentration of interleukins and its progression could be mitigated if an efficacious anti-inflammatory treatment were to be applied in an early stage of disease, as suggested in Imbimbo *et al*. [25]. Furthermore, an interesting approach might be the study the effective times of applying anti-inflammatory doses in order to complement the stress mechanism given by the UPR in lowering the production of A β -monomers and not interfering with microglia activation cycles that counteract the excess of toxic amyloid.

In this context, a possible extension of this study relies on the modelling of such treatments via an impulsive differential equation for the concentration of interleukins I (see [29,30] for a reference on this type of differential equation). This could lead to interesting optimal control problems in order to optimize both time and quantity of dose provided to mitigate AD, inspired by the work of Hu *et al*. [11]. Moreover, another important extension to the presented model is the incorporation of cell destruction due to the accumulation of oligomers in the amyloid plaques. In particular, the stress function equation (2.1) will also depend on the neural population.

One example of a possible anti-inflammatory treatment is docosahexaenoic acid (DHA). It has been demonstrated that the onset of brain diseases is linked to a deficiency in DHA, the primary omega-3 fatty acid in the brain. DHA is an essential polyunsaturated fatty acid crucial for the proper functioning of our metabolism; since it is synthesized in insufficient quantities de novo, it needs to be included in our diet (found in fatty fish or nuts). DHA is a bioactive nutrient crucial for brain development and reduces the progression of cognitive decline [31]. It also enhances synaptosomal membrane fluidity, and reduces the accumulation of A*β* peptides, fibril formation and the pro-apoptotic effects of oligomers. Note that even if diet high in omega-3 does not necessarily reflect the level of omega-3 crossing the blood–brain barrier, some studies have highlighted a more significant passage of esterified DHA in phospholipids through a specific transporter, especially in the form of structured phospholipids [32]. This form has demonstrated pro-neurogenic and anti-oxidant effects [33]. Furthermore, DHA possesses anti-inflammatory properties, which could appear as a good therapeutical hope for future research.

Concerning the dynamics of the full model incorporating the spatial dependence, the chemotaxis of microglial cells and the whole polymerization process of proto-oligomers are far from being fully understood. For the whole and complete model, we expect a similar phenomenon of hysteresis to the one observed in the spatial-homogeneous simplified model, though the analysis to prove the existence of steady states becomes way more challenging.

## Data Availability

Codes are available in the Dryad Data Repository [34].
